# Connexin 43 in Mitochondria: What Do We Really Know About Its Function?

**DOI:** 10.3389/fphys.2022.928934

**Published:** 2022-07-04

**Authors:** Kerstin Boengler, Luc Leybaert, Marisol Ruiz-Meana, Rainer Schulz

**Affiliations:** ^1^ Institute of Physiology, Justus-Liebig University, Giessen, Germany; ^2^ Department of Basic and Applied Medical Sciences—Physiology Group, Faculty of Medicine and Health Sciences, Ghent University, Ghent, Belgium; ^3^ Cardiovascular Diseases Research Group, Vall d’Hebron Institut de Recerca (VHIR), Vall d’Hebron Hospital Universitari, Barcelona, Spain

**Keywords:** connexin, mitochondria, hemichannel, ischemia-reperfusion injury, preconditioning, GJA1-20k

## Abstract

Connexins are known for their ability to mediate cell-cell communication *via* gap junctions and also form hemichannels that pass ions and molecules over the plasma membrane when open. Connexins have also been detected within mitochondria, with mitochondrial connexin 43 (Cx43) being the best studied to date. In this review, we discuss evidence for Cx43 presence in mitochondria of cell lines, primary cells and organs and summarize data on its localization, import and phosphorylation status. We further highlight the influence of Cx43 on mitochondrial function in terms of respiration, opening of the mitochondrial permeability transition pore and formation of reactive oxygen species, and also address the presence of a truncated form of Cx43 termed Gja1-20k. Finally, the role of mitochondrial Cx43 in pathological conditions, particularly in the heart, is discussed.

## 1 Introduction

Gap junction structures were first discovered in cardiac and liver cells more than half a century ago (Revel and Karnovsky, 1967; Brightman and Reese, 1969) and the connexin proteins they are composed of a few years later (Goodenough, 1974). With these findings came the insight that gap junction channels, the crucial channel for intercellular impulse conduction in the heart, are composed of two hemichannels interacting with each other. Connexin-43 (Cx43) is the main connexin in ventricular cardiomyocytes; it is synthesized in the endoplasmic reticulum, oligomerized in the Golgi apparatus and delivered to the sarcolemma as closed hemichannels. From there, hemichannels migrate towards the cell ends where they interact with their counterparts on an adjacent cell to form gap junction channels that are inserted at the rim of junctional plaques located at the intercalated disc (Gaietta et al., 2002; Rhett and Gourdie, 2012; Rhett et al., 2013). As such, sarcolemmal free hemichannels not included in gap junction channels stay closed while those in gap junctions are open. Recent work demonstrates that free Cx43-based hemichannels can also open in response to various disease-related conditions including metabolic inhibition (John et al., 1999; Kondo et al., 2000; Wang et al., 2013), cardiac ischemia (Hawat et al., 2010; Hawat et al., 2012; Wang et al., 2013), Plakophilin-2-deficient hearts as a model for arrhythmogenic cardiomyopathy (Kim et al., 2019) and in Duchenne Muscular Dystrophy hearts challenged by β-adrenergic stress (Lillo et al., 2019; Himelman et al., 2020). Furthermore, detailed evidence is available showing that hemichannels in ventricular cardiomyocytes from various species, including humans, display highly increased opening when exposed to caffeine or cell stressors like β-adrenergic stimulation, rapid pacing or combined β-adrenergic stimulation/rapid pacing (De Smet et al., 2021; Lissoni et al., 2021). These studies reported that hemichannel opening activity was most prominent at the cell ends, close to the intercalated discs, and involved co-localization of Cx43 with dyadic ryanodine type-2 receptors (RyR2)/Cav1.2 Ca^2+^ channels forming calcium (Ca^2+^) signaling microdomains that, in concert with Cx43/RyR2 protein-protein interactions, jointly control hemichannel opening. Hemichannel opening during diastole results in a substantial electrical depolarization effect due to the entry of sodium ions (Na^+^) and Ca^2+^ *via* highly conductive Cx43-based hemichannels (∼220 pS single channel conductance), but the strongest impact was observed at the level of Ca^2+^ signaling. Hemichannel opening in the vicinity of the intercalated discs was highly linked to subsequent intracellular Ca^2+^ waves that started at the cell ends where Cx43 co-localization with dyads is maximal (De Smet et al., 2021). These Ca^2+^ waves invariably resulted in delayed afterdepolarizations mediated by Ca^2+^ extrusion in exchange for Na^+^ entry by electrogenic Na^+^/Ca^2+^ exchange. Afterdepolarizations resulted in triggered action potentials, and both were effectively suppressed by Gap19, a peptide inhibitor of Cx43-based hemichannels that does not inhibit gap junctions (Wang et al., 2013). Taken together, this work demonstrates that hemichannel opening and the ensuing electrical and Ca^2+^ consequences, had a significant impact on cardiomyocyte functioning.

As can be inferred from the preceding, Cx43 hemichannels are not only present in the sarcolemma but also in organelles such as the Golgi apparatus during trafficking (Peracchia, 2020), and most importantly in the present context, also in mitochondria, in particular those just below the sarcolemma known as subsarcolemmal mitochondria of ventricular cardiomyocytes. Here, we review the role and possible function of connexins and their channels in mitochondria. The focus is on cardiac cells, in particular the highly differentiated ventricular cardiomyocytes, but data form other cells, especially endothelial cells from various vascular beds, is integrated to increase insight and complete the picture. Connexins are extremely important players in heart and brain but they come with some complexity in that they have a large repertoire of functions related to gap junction channels, hemichannels and non-channel functions of the large connexin interactome network. Despite the challenges of this complexity, the interest for hemichannels, the intermediate partner between the protein level and full-fledged gap junctions, is growing. Novel approaches and insights will be necessary to fully understand the role of these channels in the highly compartmentalized and connected environment of mitochondria.

## 2 Presence of Connexins Within Mitochondria

Whereas the localization of connexins to the plasma membrane is not in dispute it has been investigated whether the presence of the proteins is restricted to the plasma membrane or whether the proteins are also present at other intracellular structures including mitochondria. First data pointing to a mitochondrial localization of Cx43 in human umbilical vein endothelial cells were published in 2002 by Li et al. (2002). Here, the authors demonstrate co-localization of Cx43 with MitoTracker using confocal microscopy. In addition, Western Blot analysis shows Cx43 immunoreactivity in isolated mitochondria as well as in the heavy membrane fraction, which contains mitochondrial proteins. In the further course, other groups confirmed the presence of Cx43 within mitochondria of endothelial cells of various origin (Mohammad and Kowluru, 2011; Trudeau et al., 2012; Ma et al., 2020; Sankaramoorthy and Roy, 2021). Furthermore, Cx43 is detected within mitochondria isolated from stem cell antigen-1^+^ (Lu et al., 2010) and H9c2 cells (Tu et al., 2017; Pecoraro et al., 2021), from brown adipose tissue (Kim et al., 2017), from astrocytes (Kozoriz et al., 2010) and brain (Azarashvili et al., 2011) as well as from liver (Li et al., 2021). Most studies, however, focused on the mitochondrial localization of Cx43 in the heart (Boengler et al., 2005; Gorbe et al., 2011; Srisakuldee et al., 2014; Shan et al., 2015; Gadicherla et al., 2017; Wang et al., 2019; He et al., 2021; Wei et al., 2022) including neonatal cardiomyocytes (Tu et al., 2017) and embryonic stem cell-derived cardiomyocytes (Wang et al., 2021). Mitochondria from mice with inducible Cx43 knockout (Boengler et al., 2005; Gadicherla et al., 2017; Wang et al., 2019) or mice in which Cx43 was replaced by Cx32 (Miro-Casas et al., 2009) served as respective negative controls. In addition to the antibody-dependent detection of Cx43 in mitochondria using Western blot, confocal laser scan microscopy or immunoelectron microscopy (Boengler et al., 2005; Wang et al., 2019), also antibody-independent proteomic approaches strengthen the evidence of Cx43 localization in the organelles (Miro-Casas et al., 2009). The amount of Cx43 in mitochondria is influenced by pharmaceutical agents or certain pathological conditions. For example, a downregulation of mitochondrial Cx43 occurs in primary mouse retinal cells and rat retinal endothelial cells exposed to high glucose (Trotta et al., 2020; Sankaramoorthy and Roy, 2021). The amount of mitochondrial Cx43 in H9c2 cells increases upon treatment with cobalt chloride (Pecoraro et al., 2021), in human umbilical vein endothelial cells upon the administration of lipopolysaccharide (Ma et al., 2020) and in brown adipose tissue stimulated with the β3-receptor agonist CL316,243 (Kim et al., 2017). The conditions leading to alteration of mitochondrial Cx43 content in the heart are discussed in Sections 6, 7.

When studying cardiac mitochondria, it has to be considered that the heart contains different mitochondrial subpopulations including subsarcolemmal mitochondria (SSM) located beneath the sarcolemma and interfibrillar mitochondria (IFM) present between the myofibrils. SSM and IFM differ in structure and function with IFM showing higher respiratory and calcium retention capacities than SSM (Palmer et al., 1985; Palmer et al., 1986). The analysis of Cx43 within these mitochondrial subpopulations indicates that Cx43 is predominantly present in SSM (Boengler et al., 2009b; Sun et al., 2015; Denuc et al., 2016; Gadicherla et al., 2017; Wang et al., 2019) and there is no difference in the amount of mitochondrial Cx43 between SSM isolated from right and left ventricles (Boengler et al., 2021). The isolation of IFM requires the use of proteases, e.g., nagarse and such protease treatment may lead to the misinterpretation of results related to the mitochondrial amount of a certain protein (Koncsos et al., 2018). However, in case of Cx43, such misinterpretation was excluded by showing that the inhibition of nagarse does not enhance the mitochondrial amount of the protein. All available studies point to a predominant localization of Cx43 in SSM, regardless whether nagarse (Boengler et al., 2009b; Srisakuldee et al., 2014), trypsin (Sun et al., 2015) or proteinase K (Ruiz-Meana et al., 2014) was used for the IFM isolation.

As a protein containing transmembrane domains it is likely that Cx43 is also present in mitochondrial membranes and the detailed analysis of mitochondria sub-fractionated using digitonin shows the protein to be present in the inner membrane of mitochondria from cardiac, endothelial and stem cell antigen-1^+^ cells (Rodriguez-Sinovas et al., 2006; Lu et al., 2010; Trudeau et al., 2012; Srisakuldee et al., 2014). However, the use of a different protocol for the mitochondrial sub-fractionation (sonication, freeze/thaw cycles and sucrose gradient centrifugation) identifies Cx43 predominantly in the outer mitochondrial membrane (Goubaeva et al., 2007).

Cx43 may be oriented within mitochondrial membranes with its C-terminus directed towards the mitochondrial matrix or the intermembrane space. Data on mitochondria treated with either digitonin or triton (Miro-Casas et al., 2009) or on mitoplasts digested with different amounts of proteinase K (Boengler et al., 2009b) both suggest that the C-terminus of Cx43 may be facing the intermembrane space.

The characterization of interaction partners of mitochondrial Cx43 may help to clarify the function of the protein within the organelles. Using co-immunoprecipitation and subsequent mass spectrometry apoptosis-inducing factor, which under physiological conditions contributes to oxidative phosphorylation and anti-oxidant defense (Modjtahedi et al., 2006), and the beta-subunit of the electron-transfer protein (Denuc et al., 2016) are identified as proteins interacting with mitochondrial Cx43, and indeed a contribution of Cx43 to apoptosis (Sankaramoorthy and Roy, 2021) and respiration (Boengler et al., 2012) has been described. Also mitofilin (Boengler et al., 2018), which is important for cristae structure and morphology (Zerbes et al., 2012), associates with mitochondrial Cx43. The importance of Cx43 for mitochondrial function under physiological conditions is discussed in more detail in Section 6.

The mitochondrial localization is not specific for Cx43, since other connexins are also detected within the organelles, e.g., Cx40 in mitochondria of coronary endothelial cells (Guo et al., 2017), Cx32 and Cx26 in liver mitochondria (Azarashvili et al., 2011; Fowler et al., 2013). Similar to Cx43, Cx32 localizes to the inner mitochondrial membrane (Fowler et al., 2013).

Summary and open questions: the detection of mitochondrial connexins in a variety of cell types and tissues suggests that whenever connexin proteins are expressed, a fraction of it—around 15% for Cx43 in the heart (Boengler et al., 2005)—are located within mitochondria, most likely at the inner membrane. Its localization in the outer membrane has been suggested, but further studies are needed to prove or disprove this finding. Also, the orientation of Cx43 in the mitochondria membrane need to be evaluated in more detail. While some proteins interacting with Cx43 have been identified, more proteomic analyses are required to identify its proteomic network which subsequently may help to better understand its cellular/subcellular function(s).

## 3 Import of Cx43 Into Mitochondria

As the mitochondrial genome encodes only 13 proteins, more than 99% of the mitochondrial proteins have to be imported into the organelles (Wiedemann and Pfanner, 2017). In general, proteins that are ultimately localized in the inner membrane are identified *via* the TOM (translocase of the outer membrane) protein complex, consisting among other proteins of Tom20/22 and Tom40 and are inserted into the inner membrane by the translocase of the inner membrane complex (for detailed review on the mitochondrial protein import machinery see (Wiedemann and Pfanner, 2017; Zhao and Zou, 2021)). The chaperone heat shock protein 90 (Hsp90) binds unfolded proteins and delivers them to the TOM complex (Zanphorlin et al., 2016). Co-immunoprecipitation experiments demonstrate an interaction between Cx43 and Hsp90 (Rodriguez-Sinovas et al., 2006) and accordingly the inhibition of HSP90 using siRNA or pharmacological agents such as geldanamycin or radicicol reduce the mitochondrial Cx43 content (Rodriguez-Sinovas et al., 2006; Tu et al., 2017; Pecoraro et al., 2021). The interaction between Cx43 and Tom20 suggests the involvement of the TOM complex in the import of Cx43 into mitochondria (Rodriguez-Sinovas et al., 2006). A recent study demonstrates that the knockdown of Rictor, a component of the mammalian target of rapamycin complex 2, decreases the protein-protein interactions between Cx43 and Hsp90 as well as Tom20 and accordingly reduces the mitochondrial level of Cx43 in embryonic stem cell-derived cardiomyocytes (Wang et al., 2021).

Summary and open questions: data showing interactions between Cx43, the cytosolic chaperone Hsp90 and proteins of the TOM complex, which acts as the main entry gate controlling mitochondrial protein import, limits the available information to the initial steps of the Cx43 transport process towards the inner membrane. Whether the inhibition of mitochondrial protein import specifically interferes with the Cx43 amount at the inner mitochondrial membrane is currently unknown. No studies have been conducted so far to characterize the export and/or degradation of mitochondrial Cx43.

## 4 Do Connexins Form Channels Within Mitochondrial Membranes?

The well-known formation of gap junctions or hemichannels by six Cx43 monomers is the basis for the hypothesis that the protein could also form channel-like structures in the inner mitochondrial membrane. The best method for detecting such channel structures would be patch-clamp experiments on isolated mitoplasts, i.e., mitochondria in which the outer membrane has been removed (Bednarczyk et al., 2021). So far, however, no patch-clamp data on Cx43-formed channels in mitoplasts are available. Instead, putative Cx43-formed channel-like structures within mitochondria are investigated by indirect methods. For example, the reconstitution of mitochondrial Cx43 in lipid bilayers leads to the formation of functional channels (Gadicherla et al., 2017). In addition, cross-linking of mitochondrial proteins reveals Cx43-formed protein complexes corresponding to the molecular weight of a Cx43 hexamer (Miro-Casas et al., 2009) and also native gel electrophoresis suggests the presence of mitochondrial Cx43 hemichannels (Gadicherla et al., 2017). Important data strengthening the presence of Cx43-formed mitochondrial channels comes from the analysis of mitochondrial dye uptake showing that the mitochondrial Lucifer yellow uptake decreases in organelles devoid of Cx43 (Miro-Casas et al., 2009). Also, the entry of potassium ions into mitochondria in either permeabilized cardiomyocytes (Miro-Casas et al., 2009) or isolated mitochondria (Kozoriz et al., 2010; Boengler et al., 2013) is reduced when Cx43-deficient cells or organelles are studied. The putative orientation of Cx43 within the inner membrane with its C-terminus facing the intermembrane space suggests that mitochondrial Cx43-formed hemichannels may be open. Indeed, in this configuration, the “extracellular” loops in the matrix compartment are exposed to the very negative mitochondrial membrane potential (−150 to −180 mV) while the C-terminal side is exposed to the −70 to −80 mV membrane potential, corresponding to a net difference of +70 to +110 mV, which is largely above the threshold for voltage-dependent opening of Cx43-based hemichannels (+40 to + 50 mV) in ventricular cardiomyocytes (Wang et al., 2012; Gadicherla et al., 2017). As such, open hemichannels may facilitate Ca^2+^ entry into the matrix upon cytoplasmic Ca^2+^ elevation that can be blocked by hemichannel inhibition with Gap19, as experimentally demonstrated (Gadicherla et al., 2017). In addition to electrical potential, also the Ca^2+^ concentration at both sides of the inner mitochondrial membrane may influence the opening state of Cx43 hemichannels. Mitochondrial Ca^2+^ in the matrix is normally very low (∼100 nM) and increases during each contraction cycle to the hundreds of micromolar range. Low Ca^2+^ exposure of the extracellular loop side of the hemichannels is known to open the channels starting from a concentration in the range of below ∼400 µM on average (Leybaert et al., 2017). Moreover, also cytoplasmic Ca^2+^ may open the hemichannels, as is the case for sarcolemmal hemichannels (De Smet et al., 2021; Lissoni et al., 2021) but the conditions under which this could occur for mitochondrial hemichannels are currently uncharted. While most information is available for Cx43-based hemichannels, indirect evidence suggests that Cx40 also affects the mitochondrial Ca^2+^-contents. Data from mouse coronary endothelial cells demonstrate reduced mitochondrial Ca^2+^-contents in Cx40-knockout and increased amounts of mitochondrial Ca^2+^ upon overexpression of Cx40 (Guo et al., 2017).

Summary and open questions: although there is no definitive evidence for the presence of connexin-formed channels within mitochondria, the available data suggest that Cx43 and Cx40 are capable of building channel-like structures within the inner mitochondrial membrane. Alternatively, Cx43 could interact with other mitochondrial ion channels/transporters modifying their open probability/activity; such interactions need to be further scrutinized in cross-linking/mass spectrometry analyses.

## 5 Phosphorylation of Mitochondrial Cx43

**FIGURE 1:**
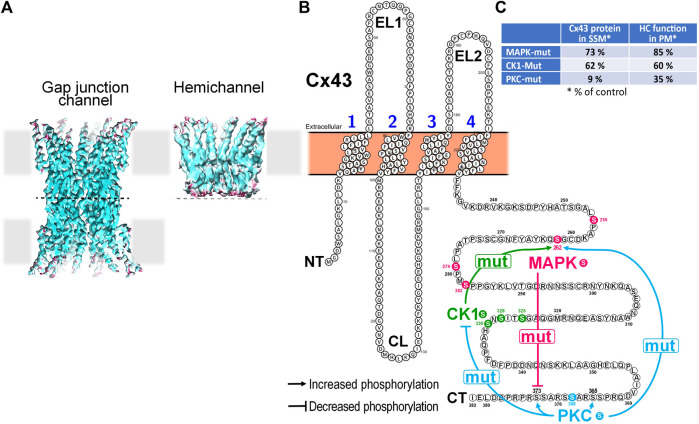
Impact of mutation of Cx43 phosphorylation sites targeted by MAPK, CK1 or PKC on the phosphorylation state of non-targeted residues in mitochondrial Cx43 and its consequences for hemichannel function and mitochondrial Cx43 presence in cardiomyocytes. **(A)** Cryo-EM reconstructions of a Cx26-based gap junction channel and hemichannel (3.5 to 4.2 Å resolution). Hemichannels are hexameric channels composed of six subunits, while a gap junction channel results from the head-to-head docking of two hemichannels on neighboring cells. (Illustration from (Khan et al., 2021)). **(B)** Membrane topology of a Cx43 monomer illustrating cross-effects of mutation of selected Cx43 amino acid residues on other phosphorylation sites within the same protein. Mutation of the 4 MAPK Ser residues to Ala (mut) reduces phosphorylation at S373, a residue targeted by Akt/PKB. Mutation of S325A, S328Y, and S330A of CK1 increases the phosphorylation at S262, a residue targeted by MAPK. PKC S368A mutation had most extensive effects. It reduced phosphorylation at all 3 sites targeted by CK1 but enhanced phosphorylation at S262 targeted by MAPK, S365 that is indirectly targeted by PKA and S373 targeted by Akt/PKB. **(C)** Table summarizing the impact of mutation of Cx43 phosphorylation sites targeted by MAPK, CK1 or PKC on the presence of Cx43 protein in SSM and the function of hemichannels as assessed in the plasma membrane (PM).

Gap junctional Cx43 is phosphorylated at several residues (at least 19 serine and two tyrosine residues have been identified so far) and thereby influence Cx43 forward transport, channel function and internalization (Solan and Lampe, 2018). Various kinases including mitogen-activated protein kinase (MAPK), protein kinase B, protein kinase C (PKC), Src and casein kinase 1 are known to phosphorylate Cx43 (Solan and Lampe, 2018). The phosphorylation of Cx43 may not be restricted to the sarcolemmal fraction of the protein and several studies have aimed to characterize the phosphorylation status of mitochondrial Cx43 specifically. Under physiological conditions, Western blot analysis on cardiac mitochondria demonstrates immunoreactive signals at molecular weights ranging between 41 and 46 kDa similar to what is observed for gap junctional Cx43 (Wang et al., 2019), thereby pointing to multiple Cx43 phosphorylation sites within mitochondria. When using antibodies against specific Cx43 phosphorylation sites, Western blot data indicate the phosphorylation of cardiac mitochondrial Cx43 at serine (S) S262 (Srisakuldee et al., 2014; Tu et al., 2017), S325/328/330 (Hirschhäuser et al., 2021), S365 (Hirschhäuser et al., 2021), S368 (Penna et al., 2009; Boengler et al., 2011; Srisakuldee et al., 2014; Shan et al., 2015), and S373 (Hirschhäuser et al., 2021) illustrated in Figure 1. Hereby, a mitochondrial fraction of PKCε may mediate the phosphorylation of Cx43 at S262 (Srisakuldee et al., 2014). In addition, mitochondrial Cx43 is phosphorylated at S262 in H9C2 cells (Waza et al., 2014). Within rat brain mitochondria, immunoreactive signals are displayed for Cx43 phosphorylated at S368 (Azarashvili et al., 2011).

The analysis of left ventricular mitochondria of mice in which S255/262/279/282 (Cx43^MAPKmut^), S325/328/330 (Cx43^CK1mut^) or S368 (Cx43^PKCmut^) are mutated to non-phosphorylatable amino indicates altered Cx43 phosphorylation at residues not targeted by the mutations (Hirschhäuser et al., 2021). Mitochondria of Cx43^MAPKmut^ mice display reduced S373 phosphorylation, whereas S262 phosphorylation increases in SSM of Cx43^CK1mut^ mice. The most extensive changes in the phosphorylation pattern of mitochondrial Cx43 appear in Cx43^PKCmut^ SSM, where the phosphorylation decreases at S325/328/330 but improves at S262, S365, and S373 (Hirschhäuser et al., 2021). The alterations in the phosphorylation of Cx43 are accompanied with changes in the mitochondrial amount of the protein which is moderately reduced in Cx43^MAPKmut^ and Cx43^CK1mut^ mice (to about 73% or 62% of that in wildtype mitochondria, respectively), but severely decreased in SSM of Cx43^PKCmut^ mice (9% of that in wildtype mitochondria) (Hirschhäuser et al., 2021).

Summary and open questions: similar to gap junctional Cx43, the mitochondrial form of the protein is phosphorylated at multiple amino acids, with no phosphorylation pattern specific for mitochondrial Cx43. Data indicate that mutation in Cx43 at specific phosphorylation sites affects phosphorylation of mitochondrial Cx43 at amino acids not affected by the mutation, showing the interdependence of the mitochondrial Cx43 phosphorylation at specific residues. How exactly the phosphorylation of mitochondrial Cx43 is regulated remains unknown at present. In addition, the phosphorylation of Cx43 also affects the mitochondrial amount of the protein. Whether or not the Cx43-phosphorylation occurs within mitochondria or whether only Cx43 phosphorylated at specific residues is translocated into the mitochondria remains to be elucidated.

## 6 Function of Mitochondrial Connexins Under Physiological Conditions

The fact that connexins are ubiquitously present in the mitochondria of different cell types, in addition to cardiomyocytes, and in several species, including humans, suggests that mitochondrial localization of connexins exerts biologically meaningful effects on cell homeostasis and survival. Nevertheless, the functions of mitochondrial connexins under physiological conditions remain largely controversial. Early evidence on mitochondrial connexins’ role was inferred from observational associations that led to proof-of-concept studies using genetically modified models, in which a specific type of connexin (mainly Cx43) was ablated, overexpressed or replaced by another isoform (mainly Cx32). The observation that ventricular cardiomyocytes, the cells with the largest mitochondrial mass and highest energy consumption preferentially express Cx43, points to a role of Cx43 on mitochondrial respiration. This prediction is confirmed by experiments using cardiomyocytes from genetically-modified mice, in which either the coding region of Cx43 is replaced by that of Cx32 or Cx43 is conditionally ablated in the heart, which unambiguously demonstrates a regulatory effect of mitochondrial Cx43 on myocardial energetics. Whereas downregulation of mitochondrial Cx43 or its replacement by Cx32 reduces mitochondrial membrane potential under resting conditions (Rodriguez-Sinovas et al., 2010) and depresses complex 1-dependent respiration and ATP generation in SSM, without affecting the respiratory efficiency in IFM (devoid of Cx43) (Boengler et al., 2012), over-expression of Cx43 in HL-1 cardiomyocytes increases mitochondrial respiration rate (Boengler et al., 2012). The decrease in the mitochondrial amount of Cx43 protein in Cx43^MAPKmut^ , Cx43^CK1mut^ and Cx43^PKCmut^ mice (to about 73%, 62%, and 9% of wildtype mitochondria, respectively) also reduces mitochondrial oxygen consumption; however, whether changes in the phosphorylation status of mitochondrial Cx43 in these mice strain contributed to the observed alteration in oxygen consumption remains to be addressed (Hirschhäuser et al., 2021).

Pharmacological inhibition of Cx43 with 18-alpha-glycyrrhetinic acid or Gap27 in SSM from rat hearts results in both a reduction of ADP-stimulated oxygen consumption after the addition of substrates to feed the respiratory complex I and less ATP generation (Boengler et al., 2012). The mechanisms by which mitochondrial Cx43 influence respiration are not clear, and several possibilities have been proposed, including the direct interaction of the monomeric Cx43 protein with the respiratory complex 1 (Boengler et al., 2012) or the Cx43-dependent activation of signaling transduction pathways that eventually regulate mitochondrial function (Waza et al., 2014). The possibility that distinct forms of Cx43 (i.e., monomeric, oligomeric and truncated, see Section 8) coexist at the mitochondrial level with different mechanisms of action cannot be ruled out. Nevertheless, the concept that Cx43 may form hemichannels within mitochondrial membranes is supported by solid and independent experimental findings both structural and functional (see Section 4). Thus, *in situ* cross-linking followed by Western blot analysis of the presence of Cx43 in the different oligomeric forms, as well as native electrophoresis gels experiments, disclosed the presence of Cx43 hexamers in intact mitochondria of rats and mice (Miro-Casas et al., 2009; Gadicherla et al., 2017). By using *in vitro* experiments of lipid bilayer reconstitution with purified and enriched Cx43, hemichannel activity was demonstrated by the presence of single channel conductance of ∼130 pS that was sensitive to the blockers Gap19 and RRNY peptides, as detected in sarcolemmal hemichannels (Gadicherla et al., 2017). The concept that mitochondrial Cx43 forms hemichannels is strengthened by functional data, indicating that mitochondrial Cx43 hexameric structures are capable to uptake Lucifer yellow and are Ca^2+^ and K^+^ permeant (Miro-Casas et al., 2009; Kozoriz et al., 2010; Boengler et al., 2013). As the entry of potassium ions into the mitochondrial matrix has long been recognized as an important regulator of mitochondrial swelling and respiratory efficiency (Kowaltowski et al., 2001; Lim et al., 2002) it is hypothesized that Cx43-formed hemichannels in the inner mitochondrial membrane may influence mitochondrial oxygen consumption by regulating potassium fluxes. Indeed, several types of chemically unrelated Cx43 channel blockers (i.e., carbenoxolone, heptanol, 18-alpha-glycyrrhetinic acid and Gap19 peptide) exert inhibitory effects on mitochondrial potassium flux and the concept is strengthened by the fact that these pharmacological effects cannot be recapitulated in isolated mitochondria from Cx43-deficient mice (Miro-Casas et al., 2009; Boengler et al., 2013).

As for the hemichannels present in the plasma membrane, mitochondrial Cx43 hemichannels may adopt distinct conductance states that can affect mitochondrial function. Thus, whereas mitochondrial Cx43 hemichannels are expected to be closed under physiological conditions to avoid the dissipation of the intermembrane proton gradient necessary for ATP production, they may open temporarily in response to certain triggers (Boengler and Schulz, 2017) and thereby contribute to uncoupling. Compared to the unitary conductance of ∼75 pS of a typical uncoupling channel like uncoupling protein 1 (Huang and Klingenberg, 1996), Cx43 hemichannels have a larger unitary conductance of ∼220 pS in the sarcolemma (De Smet et al., 2021) and ∼130 pS as concluded from mitochondrial Cx43 reconstituted in lipid bilayers (Gadicherla et al., 2017). The number of hemichannels present in the inner mitochondrial membrane is currently unknown. Sarcolemmal recordings in cell-attached mode from mouse ventricular cardiomyocytes in the vicinity of the intercalated disc point to 1 and sometimes 2 active hemichannels for a pipette internal diameter of ∼300 nm [scanning ion conductance microscopy-based recordings, see (De Smet et al., 2021)], corresponding to 14 active hemichannels per µm^2^ (for 1 active hemichannel in pipette recording). Mitochondria contain 15% of the total Cx43 or 15/85 of the sarcolemmal Cx43. The perinexal area surrounding gap junctions has the highest hemichannel density so let’s further assume a 10-fold lower density in subsarcolemmal mitochondria; taking both factors into account gives a ∼0.25 hemichannels per µm^2^ surface area of the inner mitochondrial membrane. For a typical SSM consisting of a round sphere of ∼1 µm diameter [based on (Holmuhamedov et al., 2012), Figure 1], the inner membrane surface area can be estimated to be ∼15 µm^2^ including the cristae (based on a density of 30 µm^2^/µm³). Taken together, these estimates yield ∼4 hemichannels per mitochondrion as a crude estimate. Given their high conductance and large driving force for current flow provided by the mitochondrial membrane potential, this would certainly be enough to impact mitochondrial function and uncoupling. Obviously, the true impact will depend on the hemichannel open time, a factor that is currently unknown and not included in the current estimation.

The occurrence of mild mitochondrial uncoupling secondary to changes in Cx43 hemichannels may have consequences on reactive oxygen species (ROS) generation (Cadenas, 2018) and mitophagy (He et al., 2020). Indeed, isolated cardiomyocytes from Cx43-deficient mice become resistant to protection by the ATP-dependent potassium channel opener diazoxide due to impaired mitochondrial ROS production (Heinzel et al., 2005), whereas induction of Cx43 hemichannel permeability by nitric oxide donors increases ROS production in cardiac SSM (Soetkamp et al., 2014). Paradoxically, in an *in vitro* model of H9C2 cells exposed to chemically-induced hypoxia, diazoxide attenuates ROS production and exerts an antiapoptotic effect as long as mitochondrial Cx43 is present (Pecoraro et al., 2021). The phosphorylation state of mitochondrial Cx43 (either by dephosphorylation or hyperphosphorylation) can modulate mitochondrial apoptotic pathway in neonatal rat ventricular cardiomyocytes (Fu et al., 2021b). Similarly, in human umbilical vein endothelial cells, lipopolysaccharide-induction of oxidative stress and apoptosis are associated with an increased level of both total and phosphorylated mitochondrial Cx43, whereas Cx43 hemichannel blockade with Gap19 has a protective effect against ROS production and cell survival (Ma et al., 2020). Nevertheless, the pathophysiological relevance of these evidences in a more relevant context and their potential contribution to other mitochondrial physiological parameters (i.e., mitophagy, dynamics) remain to be addressed. Mitochondrial Cx43 hemichannels have also been shown to be calcium-permeable and contribute to mitochondrial calcium homeostasis (Gadicherla et al., 2017). Importantly, the contribution of mitochondrial Cx43 to calcium fluxes may modulate the cell death susceptibility through opening of the mitochondrial permeability transition pore (mPTP) (Gadicherla et al., 2017). Mitochondrial PTP is a large conductance pore whose persistent opening dissipates the intermembrane proton gradient necessary to sustain oxidative phosphorylation (Ong et al., 2015). Opening of mPTP allows water to enter to the mitochondrial matrix, leading to mitochondrial swelling, energy collapse and eventually mitochondrial membrane rupture and cell death (Kwong and Molkentin, 2015). As such, it has been shown to contribute to pathophysiological signaling during the first minutes of reperfusion, due to concurrent effects of calcium and ROS as mPTP triggers (Di Lisa and Bernardi, 2015). Although the molecular entity of mPTP remains controversial, some experimental data suggest that mitochondrial ATP synthase (either in its monomeric form or as a consequence of defective dimerization) could form the ultra high conductance (order of 1,000 pS) channel (Giorgio et al., 2013; Alavian et al., 2014; Bonora et al., 2017; Urbani et al., 2019). In this context, it has been demonstrated that fibroblast growth factor 2 stimulated mitochondrial Cx43 phosphorylation at PKCε target sites and increased the tolerance for calcium-induced mPTP opening (Srisakuldee et al., 2014). However, in another study, the mutation of the PKC-target site S368 to alanine seemed to be without consequences for calcium-induced mPTP opening, at least under control conditions (Hirschhäuser et al., 2021). Nevertheless, blocking Cx43 hemichannels with Gap27 reduced the tolerance for calcium-induced mPTP opening, indicating that mitochondrial hemichannel function is required for fibroblast growth factor -related protection (Srisakuldee et al., 2014). In contrast, under conditions of ischemia-reperfusion (IR), mitochondrial Cx43 hemichannel opening has been demonstrated to enhance mitochondrial calcium entry, leading to increased cell death that is counteracted by hemichannel block with RRNYRRNY peptide, which acts more potently on mitochondrial hemichannels compared to Gap26 or Gap19 (Gadicherla et al., 2017).

Recently, it has been demonstrated in ventricular cardiomyocytes that sarcolemmal Cx43 hemichannel opening is linked to activation of RyR2 (De Smet et al., 2021; Lissoni et al., 2021), which brings up the question whether mitochondrial hemichannels could perhaps also be influenced by and synchronized to ryanodine receptor activation with each contraction cycle. However, the ryanodine receptor-hemichannel link is not just a calcium signaling link but also involves more direct molecular interactions between Cx43 and RyR2s. As such, the fact that mitochondrial Cx43 hemichannels localize at the inner mitochondrial membrane and are not present in IFM, which are in close contact with sarcoplasmic reticulum, suggests that their functional regulation may be independent of their anatomical proximity to other organelles.

**FIGURE 2:**
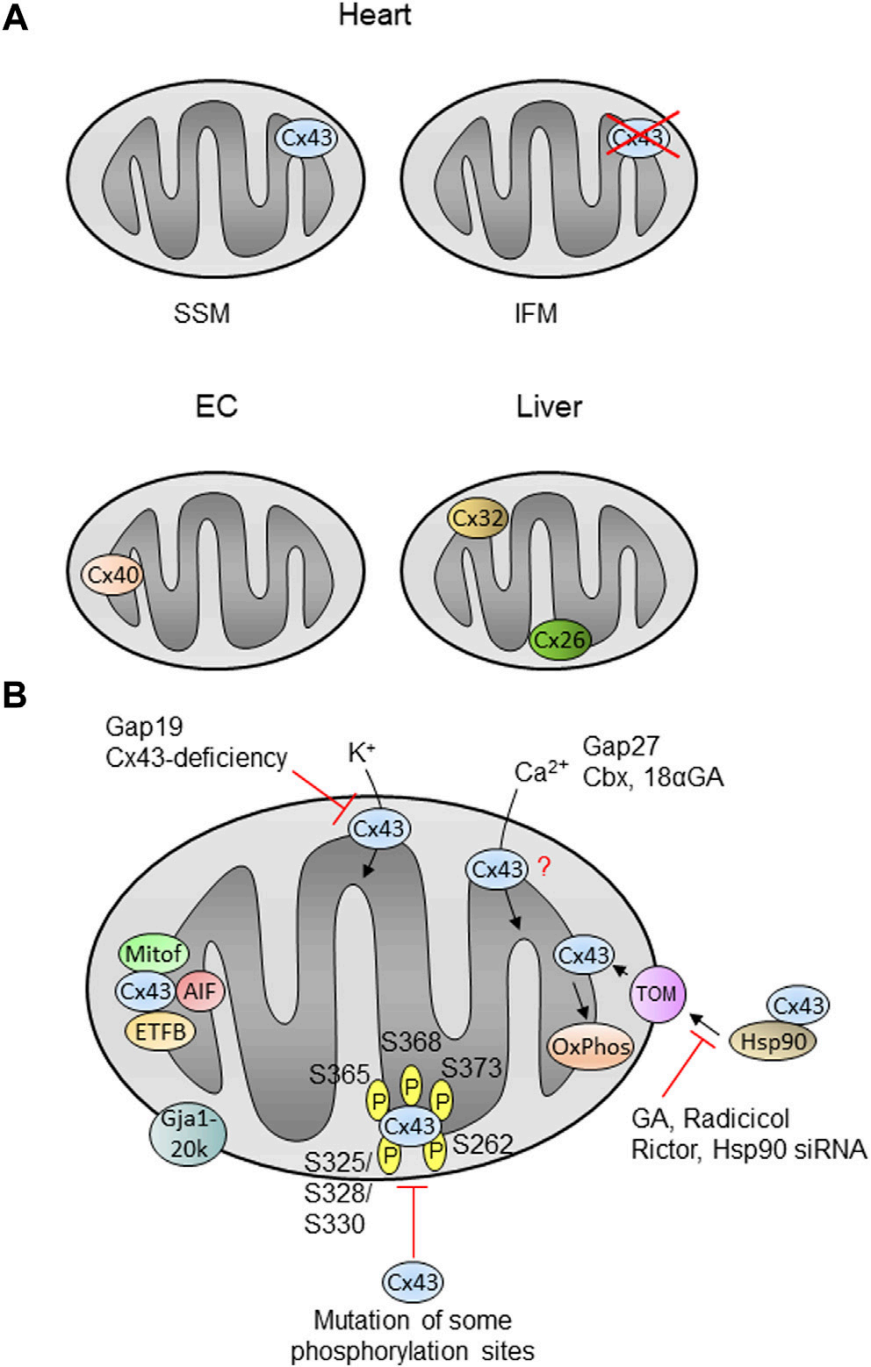
Mitochondrial localization and function of connexins in cells or organs. **(A)** The localization and function of mitochondrial Cx43 is best described in the heart, where the protein is present in SSM (subsarcolemmal mitochondria), but is nearly absent in interfibrillar mitochondria (IFM). Cx43 is also detected in mitochondria of other cells or organs (see text). Cx40 is found in mitochondria of endothelial cells (EC), whereas liver mitochondria contain Cx32 and Cx26. **(B)** Cx43 is imported into cardiac SSM in a pathway involving Hsp90 (heat shock protein 90) and TOM proteins (translocase of the outer membrane) and is finally inserted into the inner membrane. Inhibition of Hsp90 using geldanamycin (GA), radicicol or Hsp90 siRNA as well as Rictor decreases the mitochondrial Cx43 amount. The amino acids known to be phosphorylated within Cx43 are indicated by ℗. Mutation of some phosphorylation sites decreases the mitochondrial Cx43 amount. Proteins known to interact with mitochondrial such as mitofilin (Mitof), apoptis-inducing factor (AIF) or the beta-subunit of the electron transferprotein (ETFB) are shown. Mitochondrial Cx43 presumably forms hemichannels and the inhibition of such channels using Gap19 or by Cx43-deficiency decreases the potassium flux into the organelles. Calcium ion (Ca^2+^) uptake is modified in the presence of the inhibitors Gap27, carbenoxolone (Cbx) or 18α-glycyrrhetinic acid (18αGA), however, the exact role of mitochondrial Cx43 in this process is unclear (?). Cx43 influences oxidative phosphorylation (OxPhos). The N-terminally truncated form of Cx43, Gja1-20k, localizes at the outer mitochondrial membrane. |− : inhibition; for further details see text.

A summary of the mitochondrial connexin localization and function is presented in Figure 2. Summary and open questions: Mitochondrial Cx43, either through interaction with target proteins or through the formation of hemichannels, appears to play several independent roles in some key mitochondrial parameters, including the regulation of the rate of oxygen consumption, ROS generation and calcium homeostasis in the mitochondrial matrix. Whether minimal decreases in the amount of mitochondrial Cx43 (∼25%) are sufficient to explain the decreases in respiration and whether Cx43 phosphorylation is a main determinant of mitochondrial function needs to be addressed in further studies. Although Cx43 is not the only connexin isoform present in the mitochondria (see Section 2), the relative contribution of other connexin isoforms to the regulation of mitochondrial function in the different tissues and organs is currently unknown.

## 7 Pathophysiological Relevance of Mitochondrial Connexins

Changes in connexin’s abundance, distribution, posttranslational modifications and degradation are known to play an important pathophysiological role in several conditions and organs, particularly in the heart and the brain (Rodríguez-Sinovas et al., 2012; Huang et al., 2021; Rodríguez-Sinovas et al., 2021; Tittarelli, 2021). The specific role of mitochondrial connexins in these contexts is less known. In the heart, mitochondrial Cx43 has been linked to IR injury and the cardioprotective effect of some interventions (Ruiz-Meana et al., 2008), although the cause-effect relationship is more difficult to establish. This is because whatever strategy is used to inhibit Cx43 hemichannels (pharmacological blockade or genetic ablation) has a beneficial effect against IR injury by mechanisms that also involve sarcolemmal hemichannels (Rodríguez-Sinovas et al., 2007; Rodriguez-Sinovas et al., 2010; Wang et al., 2013), making it difficult to dissect the specific contribution of mitochondrial Cx43. After myocardial IR, the efficiency of mitochondria to restore their respiratory function and ATP synthesis, and to contribute to ROS generation and calcium homeostasis are all factors directly involved in cell death/survival (Garcia-Dorado et al., 2012; Davidson et al., 2020), and mitochondrial Cx43 has been shown to modulate them to varying degrees, which highlights its role as a therapeutic target. In this context, the robust and highly reproducible protective effect against IR-induced cell death afforded by ischemic preconditioning [(IPC), i.e., brief cycles of transient ischemia before an index ischemia] enhances the translocation of Cx43 to mitochondria in the hearts of mouse, rats and pigs (Boengler et al., 2005; Rodriguez-Sinovas et al., 2006; Sun et al., 2015). Similarly, the reduction of infarct size induced by pharmacological preconditioning with diazoxide (an agonist of mitochondrial ATP-dependent potassium channels) increases the amount of mitochondrial Cx43 in murine hearts (Rodriguez-Sinovas et al., 2006), suggesting a causative relationship between mitochondrial Cx43 import and the benefits exerted by such cardioprotective interventions. Nevertheless, whereas the cardioprotective effect of diazoxide is abrogated by the pharmacological inhibition of Cx43 import to mitochondria, that of IPC is not (Rodriguez-Sinovas et al., 2006). An explanation for this discrepancy could be the redundant cardioprotective pathways triggered by the ischemic conditioning strategies (Hausenloy et al., 2016), unlike the more specific mechanisms activated by pharmacological agonists. Thus, diazoxide’s beneficial effect appears to depend on the stimulation of ROS generation by a mitochondrial Cx43-associated mechanism, as inferred from the fact that cardiomyocytes isolated from Cx43^+/−^ mice failed to produce mitochondrial ROS in response to diazoxide and were resistant to be protected against IR injury (Heinzel et al., 2005). For IPC protection, mitochondria are important mediators through several mechanisms, including the increased potassium permeability and ROS generation secondary to enhanced S-nitrosation of mitochondrial Cx43 (Sun et al., 2015). However, IPC can have a direct beneficial effect on mitochondrial function that goes beyond cytosolic signaling pathways and still involve Cx43 hemichannels, as indicated by *in vitro* IR experiments using isolated mitochondria from wild-type mice, in which IPC exerted a protective effect on the recovery of the respiratory function even in the absence of cytosolic components, but it lost its therapeutic efficacy in mitochondria isolated from Cx43-deficient mice (Ruiz-Meana et al., 2014). Regarding the protection afforded by ischemic postconditioning [(IPostC), i.e., brief cycles of transient ischemia during the initial minutes of reperfusion], the contribution of mitochondrial Cx43 is not well defined. Some studies indicate that unlike IPC, IPostC-mediated cardioprotection is preserved in Cx43-deficient mice (Heusch et al., 2006). Other evidences indicate changes in the amount of phosphorylated mitochondrial Cx43 after IR in response to IPostC (Boengler et al., 2011) paralleled by more preserved mitochondrial structure and integrity (Penna et al., 2009). Prevention of Cx43 translocation to mitochondria in H9c2 cells by siRNA targeting to HSP90 abrogates the effect of hypoxic postconditioning on ROS generation and cell viability (Tu et al., 2017).

In other cardiac conditions, like arrhythmias and heart failure, in which the level and distribution of plasma membrane Cx43 protein has an indisputable pathophysiological role, the data on the specific contribution of mitochondrial Cx43 is scarce and rather descriptive in nature. Thus, the amount of mitochondrial Cx43 and phospho-Cx43 (S368) was reported to be reduced in a rat model of furazolidone-induced dilated cardiomyopathy, although the mechanistic effect of this reduction is not clear (Shan et al., 2015). More recently, translocation of Cx43 to mitochondria has been proposed to attenuate the cardiotoxicity induced by some antineoplastic drugs. Thus, pharmacological inhibition of Cx43 import to mitochondria with radicicol increased the doxorubicin-induced oxidative and nitrosative stress in H9c2 rat myoblasts (Pecoraro et al., 2015; Pecoraro et al., 2020a) and aggravated the mitochondrial damage secondary to trastuzumab treatment in these cells, favoring apoptotic death (Pecoraro et al., 2020b). While these effects are important, their relevance in more physiological models deserves further investigation.

The potential therapeutic value of mitochondrial Cx43 has been shown in cells other than cardiomyocytes. In the brain, IR injury is associated with a reduction of both total mitochondrial Cx43 and the phosphorylated fraction of mitochondrial Cx43 (Hou et al., 2016). Importantly, the IR-induced deleterious effects on cell survival could be partially prevented by diazoxide activation of PKC-dependent pathway, which in turn attenuated the reduction of mitochondrial Cx43, pointing to a correlation between preserved mitochondrial Cx43 level and the neuroprotective effect of some strategies (Hou et al., 2016). Similarly, the harmful effects of the parkinsonian toxin MPP+ on human dopaminergic SH-SY5Y cells are attenuated by over-expressing Cx43, an intervention that ultimately protects mitochondrial function and integrity and reduces mitochondrial-driven apoptotic death (Kim et al., 2016). In bone marrow stem cells, the specific overexpression of mitochondrial Cx43 has been reported to mimic the cytoprotective effect of IPC on cell survival (Lu et al., 2010) and the study emphasizes the importance of mitochondrial Cx43 in the protection by IPC apart from its other cellular localizations. In rat retinal endothelial cells, hyperglycemic conditions (simulating diabetic retinopathy) reduce mitochondrial Cx43 hemichannels permeability and increase mitochondrial fragmentation and apoptosis (Trudeau et al., 2012). Mitochondrial Cx43 can have additional still non explored pathophysiological roles through its transient and reversible interaction with other proteins, including apoptosis-inducing factor and the beta-subunit of the electron-transfer protein, involved in oxidative phosphorylation and redox control (Denuc et al., 2016), mitofilin (Boengler et al., 2018) that participate in mitochondrial inner folding and cristae architecture and the proapoptotic Bax (Sun et al., 2012). Indeed, the interaction of mitochondrial Cx43 with Bax has been suggested to have a protective tumor-suppressive effect in pancreatic cancer cells (Sun et al., 2012).

**FIGURE 3:**
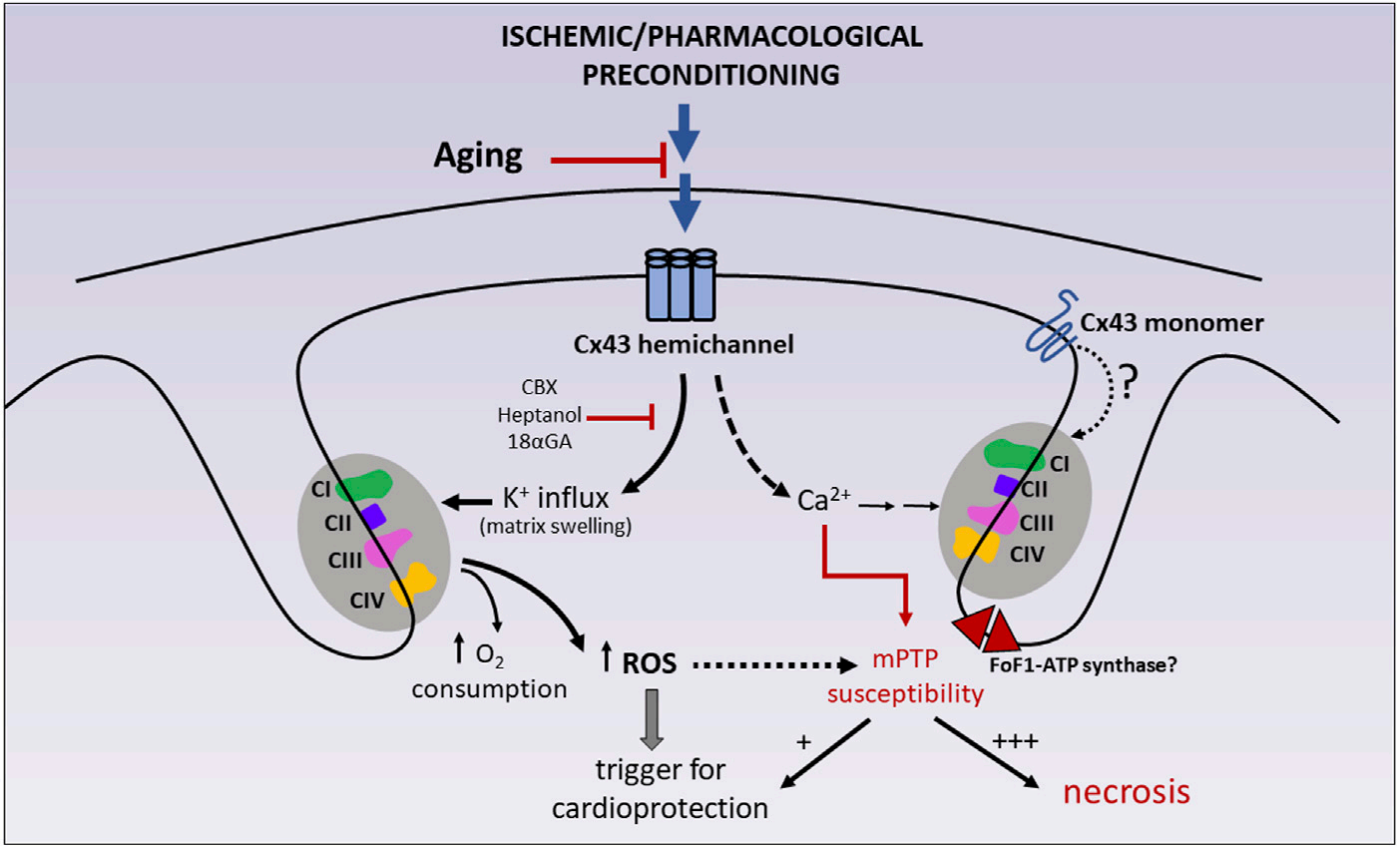
Participation of mitochondrial Cx43 on cardioprotection and tolerance to IR injury. Ischemic preconditioning (IPC) and some pharmacological preconditioning strategies (diazoxide) have been shown to reduce cell necrosis after IR injury by stimulating mitochondrial K^+^ entry *via* Cx43 hemichannels and, concomitantly, mitochondrial respiration and ROS generation. Due to the absence of direct patch clamp studies on Cx43-formed channels in mitoplasts (i.e., mitochondria in which the outer membrane has been removed), the possibility that monomeric Cx43 also plays a role in the activation of the respiratory enzymes cannot be ruled out. Transient ROS production acts as a trigger of cardioprotective signaling. These effects can be abrogated by chemically unrelated hemichannel blockers, like heptanol, carbenoxolone (CBX) and 18-alpha-glycyrrhetinic (18αGA). Mitochondrial Cx43 hemichannels are also suggested to be calcium permeable, therefore participating in the homeostasis of mitochondrial calcium, a well-known sensitizer of mPTP opening. Whereas transient mPTP (+) may itself participate in preconditioning protection, sustained mPTP (+++) uncouples mitochondrial respiration and precipitates mitochondrial swelling, membrane rupture and cell necrosis. CI-CII-CIII-CIV: mitochondrial respiratory complexes (I–IV). |− : inhibition; for further details see text.

During physiological aging, there is a decline in Cx43 expression in different cell types, organs and animal species (Boengler et al., 2006). In the heart, this age-associated reduction of Cx43 has been described both at the gap junctions (Chen and Jones, 2000; Jones et al., 2004) and in mitochondrial membranes (Boengler et al., 2007). Interestingly, the aged heart becomes more resistant to the protective effect of some conditioning strategies and cardiomyocyte death is aggravated during IR injury in preclinical models of aging animals (Boengler et al., 2009a; Ruiz-Meana et al., 2020). The increased cardiomyocyte vulnerability to IR injury has been partially attributed to a more pronounced mitochondrial energetic failure and an impaired mitochondrial capacity to buffer cytosolic calcium overload (Fernandez-Sanz et al., 2015). Nevertheless, the causal link between the age-associated decrease in mitochondrial Cx43 content and the reduced tolerance of cardiac mitochondria to withstand an ischemic insult has not been established, nor has the loss of cardioprotection during aging. In fact, IFM (devoid of Cx43) are the most sensitive to the deleterious effect of aging, whereas respiratory capacity and calcium tolerance remain rather preserved in SSM of aged hearts (Fannin et al., 1999; Fernandez-Sanz et al., 2014). In addition, the possibility that mitochondrial Cx43 is subject to some deleterious post-translational modifications prevalent in aging, such as glycooxidative damage, and the functional consequences this may have in the heart or other organs should be investigated. The participation of mitochondrial Cx43 on cardioprotection and tolerance to IR injury is illustrated in Figure 3.

Summary and open questions: The potential of mitochondrial Cx43 as a therapeutic target has been mainly established in cardiomyocytes, in which increased translocation of Cx43 to the inner membrane and downstream activation of oxygen consumption and ROS production is causally linked to the protective effect of ischemic or pharmacologically-induced preconditioning against cell death during IR. More studies are needed in organs other than the heart and in different pathological contexts (arrhythmias, heart failure, neurodegenerative diseases, cancer). The relative contribution of post-translational modifications on mitochondrial Cx43 hemichannels permeability remains controversial. It is largely unknown to what extent mitochondrial Cx43 levels and downstream signaling can be modified by aging, sex, drugs or other physiological conditions.

## 8 Alternative Translation of Cx43

The Gja1 gene encoding Cx43 contains two exons, the second of which comprises the entire coding region (Fishman et al., 1991). The use of antibodies directed against the carboxyterminus of Cx43 in Western blot analysis detects not only the full-length protein of 43 kDa size, but also a 20 kDa fragment in the heart (Smyth and Shaw, 2013; Xiao et al., 2020; Fu et al., 2021a). This 20 kDa fragment—termed Gja1-20k—is generated by alternative translation using an in-frame AUG codon as ribosomal start site to initiate translation (Smyth and Shaw, 2013; Salat-Canela et al., 2014). Gja1-20k constitutes the entire carboxyterminus as well as a large part of the transmembrane-4 (TM4) domain.

In addition to the heart, Gja1-20k is expressed in NRK (normal rat kidney epithelial), bEnd3 (brain endothelial), and AT84 (oral epithelial) cells (Joshi-Mukherjee et al., 2007), in mesenchymal cells (James et al., 2018), in mammary gland cells (Zeitz et al., 2019), in astrocytes (Ul-Hussain et al., 2014) as well as in *Xenopus* neural crest (Kotini et al., 2018). The translation of Gja1-20k is regulated, e.g., by molecular target of rapamycin (Salat-Canela et al., 2014; Ul-Hussain et al., 2014), MAP kinase-interacting serine/threonine-protein kinases 1/2 (Salat-Canela et al., 2014) or Cyclosporin A (Joshi-Mukherjee et al., 2007). The analysis of the subcellular localization demonstrates Gja1-20k within the Golgi apparatus (James et al., 2018), the nucleus (Kotini et al., 2018) and interestingly also in mitochondria (Fu et al., 2017; Wang et al., 2019; Fu et al., 2021a; Shimura and Shaw, 2022). Similar to full-length Cx43, Gja1-20k locates for the most part to SSM (Wang et al., 2019) and unlike full-length Cx43 to the outer mitochondrial membrane (Basheer et al., 2018). Upon overexpression of Gja1-20k the phosphorylation of full-length Cx43 is reduced, however, the amount of total Cx43 remains unchanged (Ren et al., 2021). Mice specifically lacking Gja1-20k show reduced amounts of full-length Cx43 and gap junctions, develop abnormal cardiac electrical excitation and have a median lifespan of only 18 days thus demonstrating the important role of Gja1-20k in maintaining normal cardiac function (Xiao et al., 2020). The role of Gja1-20k relates to hexamer oligomerization (James et al., 2018), stabilization of actin filaments and delivery of Cx43-hexamers to the intercalated discs (Basheer et al., 2017). Concerning its influence on mitochondria, Gja1-20k acts as an organelle chaperone and assists in the microtubule-based mitochondrial transport to the cell periphery (Fu et al., 2017). Upon treatment with hydrogen peroxide Gja1-20k limits mitochondrial fragmentation and thereby helps to preserve the mitochondrial network upon oxidative stress (Fu et al., 2017). Whereas under baseline conditions the overexpression of Gja1-20k does not alter mitochondrial function of rat neonatal cardiomyocytes, Gja1-20k improves mitochondrial membrane potential and oxygen consumption and reduces ROS formation in angiotensin II-induced hypertrophy (Fu et al., 2021a). In contrast, the adenoviral overexpression of Gja1-20k in mouse hearts induces metabolic quiescence as shown by decreased membrane potential, respiration, and ROS formation, but simultaneously stimulates mitochondrial biogenesis. However, the metabolic quiescence does not lead to cardiac dysfunction (Basheer et al., 2018). Also, in a rat model of traumatic brain injury overexpression of Gja1-20k in astrocytes enhances mitochondrial biogenesis (Ren et al., 2020).

Upon hypoxia, the expression of Gja1-20k increases in the rat brain (Ul-Hussain et al., 2014). In the heart, the amount of Gja1-20k enhances both after acute and prolonged IR injury in mice and also in patients with chronic end-stage ischemic cardiomyopathy, demonstrating improved expression of Gja1-20k as a common feature of acute and prolonged ischemic injury (Basheer et al., 2018). The increased levels of Gja1-20k are not restricted to total protein extracts, but are also detected within mitochondria after IR injury, after a preconditioning stimulus (Basheer et al., 2018) and in SSM of mice undergoing post-ischemic administration of estrogen (Wang et al., 2019). Whereas dephosphorylation and lateralization of Cx43 at the plasma membrane are often induced by ischemia (Schulz et al., 2015), overexpression of Gja1-20k helps to maintain Cx43 localization at the intercalated discs in the setting of acute ischemia (Basheer et al., 2017). Gja1-20k is cardioprotective in the context of IR injury as the overexpression of alternatively transcribed Cx43 reduces myocardial infarction both *in vitro* and *in vivo* (Basheer et al., 2018). A reduced ROS formation may contribute to such cardioprotection (Basheer et al., 2018).

Summary and open questions: alternative translation generates a truncated form of Cx43 termed Gja1-20k. Gja1-20k localizes at different subcellular compartments including mitochondria and influences the amount and delivery of Cx43 to the intercalated discs as well as mitochondrial function in terms of respiration, membrane potential and mitochondrial biogenesis. Gja1-20k localizes at different subcellular compartments including mitochondria thereby demonstrating the protective potential of the Cx43 TM4-carboxyterminal protein. Whether or not Gja1-20k is suitable as a therapeutic target to increase cardiomyocyte survival after IR injury or other forms of cardiac stress will the subject of future studies.

## 9 Summary and Conclusion

Whereas the traditional view on connexins is that of proteins localized at the plasma membrane and important for cell-cell communication, recent studies point to the fact that the subcellular localizations and functions of connexins are more diverse than originally thought. The present review focuses on the presence of connexins in mitochondria and their role under physiological and pathological conditions. The secure/established facts on mitochondrial connexins, especially Cx43, and ambiguous/non-established issue are summarized in Table 1. In general, the mitochondrial localization of connexins is not restricted to a specific connexin or to a specific cell type since several connexins have been detected in mitochondria of various cells, organs and species. However, not all mitochondria of a connexin-expressing cell necessarily contain the protein, as shown by the predominant presence of Cx43 at the inner membrane of cardiac SSM, whereas IFM have only very limited Cx43-amounts. The localization of Cx43 in SSM points to a specific function of the protein in this mitochondrial subpopulation. Hsp90 contributes to the delivery of Cx43 to the TOM complex in the mitochondrial outer membrane, which then is suggested to mediate the entry of Cx43 into the organelles. However, since the translocation of Cx43 to the inner mitochondrial membrane definitely involves more proteins/protein complexes than the ones already described more research is needed to identify the exact pathways by which Cx43 or other connexins enter the mitochondria. Moreover, it is unclear whether the presence of a mitochondrial target sequence is a prerequisite for connexin import into mitochondria.

**TABLE 1:** Secured/established facts and ambiguous/not established issues on mitochondrial connexins, especially Cx43. CK1, casein kinase 1; Hsp90, heat shock protein 90; IFM, interfibrillar mitochondria; IR, ischemia/reperfusion; MAPK, mitogen-activated protein kinase; mPTP, mitochondrial permeability transition pore; PKC, protein kinase C; ROS, reactive oxygen species; S, serine; SSM, subsarcolemmal mitochondria; TOM, translocase of the outer membrane.

	Secure/established	Ambiguous/not established
Connexins generally detected in mitochondria	Cx43, Cx40, Cx26, Cx32	Other connexins; systematic analysis of mitochondrial connexin localization in cell lines, primary cells, organs
Predominant localization in SSM, not IFM	Cx43	Other connexins
Submitochondrial localization of Cx43		Inner or outer mitochondrial membrane
Import of Cx43 into mitochondria	Import in a Hsp90/TOM-dependent pathway	Detailed analysis of the pathway directing Cx43 to the inner and/or outer membrane; export/degradation of mitochondrial Cx43 and its role in pathological conditions
Cx43-formed mitochondrial hemichannels	Protein complexes corresponding to the molecular weight of hemichannels	Patch-clamp data on mitoplasts
	Functional channels upon reconstitution of mitochondrial Cx43 in lipid bilayers	Visualization of protein complex in mitochondrial membranes
	Cx43 inhibition reduces dye and potassium uptake	Open time of mitochondrial hemichannels
Phosphorylation of mitochondrial Cx43	S262, S325/328/330, S365, S373, S368	Other residues
	Mutation of specific phosphorylation sites changes the amount of mitochondrial Cx43 and the phosphorylation at other residues	Phosphorylation occurs prior to import, within mitochondria or both
	Physiological conditions: mutation of Cx43 phosphorylation sites targeted by MAPK, PKC or CK1 reduces mitochondrial oxygen consumption, no influence on ROS formation or mPTP opening	Relevance of Cx43 phosphorylation for mitochondrial function in terms of mitophagy and/or mitochondrial dynamics under physiological conditions
	Induction of apoptosis alters the phosphorylation of mitochondrial Cx43	Relevance of Cx43 phosphorylation for mitochondrial function in pathological situations
Function of mitochondrial Cx43	Reduction/inhibition of Cx43 decreases oxygen consumption, potassium uptake	Mechanism by which mitochondrial Cx43 affects respiration, calcium homeostasis and mPTP opening, ROS formation and uptake of potassium ions: via Cx43-based hemichannels or modulation of other channels Systematic analysis of proteins interacting with mitochondrial Cx43
	Cx43 influences mitochondrial calcium homeostasis, ROS formation	Influence of Cx43 for other mitochondrial parameters such as mitophagy or mitochondrial dynamics
Pathological conditions	IR injury and ischemic preconditioning: amount and CK1-mediated phosphorylation are involved	Mechanism by which mitochondrial Cx43 contributes to IR injury and the protection from it; its role in postconditioning
	Amount of mitochondrial Cx43 declines with ageing	Link between the age-associated decrease in mitochondrial Cx43 and the reduced tolerance to withstand an ischemic insult
		Contribution of mitochondrial Cx43 to heart failure, arrhythmia, neurodegenerative diseases, cancer
Gja1-20k	Gja1-20k localizes at different subcellular compartments including mitochondria, influences mitochondrial function, amount of Cx43 at the intercalated discs	Import of Gja1-20k into mitochondria, submitochondrial localization
		Mechanism by which Gja1-20k influences mitochondrial function
	Amount of Gja1-20k increases with ischemia	Mechanism by which mitochondrial Gja1-20k contributes to IR injury or other pathological conditions
	Overexpression of Gja1-20k decreases IR injury	

Similar to Cx43 at the plasma membrane, mitochondrial Cx43 is phosphorylated at several amino acids. Due to the limited availability of phospho-specific antibodies, no complete analysis of the phosphorylation status of mitochondrial Cx43 has been performed up to now and also no mass spectrometry data are available. It is unclear whether Cx43 becomes (additionally) phosphorylated within the mitochondria or whether the phosphorylation of the protein is a prerequisite for the import into the organelles, both under physiological and pathological conditions. However, under basal conditions the phosphorylation status of Cx43 is important for the mitochondrial amount of the protein and influences mitochondrial respiration. The contribution of mitochondrial Cx43 phosphorylation towards mitochondrial function under pathophysiological conditions needs to be investigated in more detail.

When conducting research on mitochondrial connexins, the obvious issue relates to the formation of channels within the inner mitochondrial membrane. Whereas the definite proof of mitochondrial connexin-formed hemichannels by patch-clamp analysis is still pending, the available data are in-line with the hypothesis that mitochondrial connexins, similar to connexins at the plasma membrane, assemble into hexamers and are capable of forming channels. The entry of potassium and calcium ions into the mitochondria *via* Cx43-formed channels could be relevant for the influence of Cx43 on mitochondrial function in terms of oxygen consumption, ROS formation and mPTP opening. Therefore, by forming mitochondrial hemichannels, connexins could regulate mitochondrial ion homeostasis, thereby be important for cellular energy supply and finally for cell survival. In the setting of IR injury, mitochondrial Cx43 is involved in the cardioprotection by ischemic and pharmacological preconditioning and may exert its protective effect *via* affecting respiration and/or ROS formation, whereas the exact site of ROS production affected by Cx43 has not yet been identified. The role of mitochondrial Cx43 in ischemic postconditioning is less established and it is unclear if the inconsistent data are related to the different experimental models (e.g., IR *in vitro* or *in vivo*) or the postconditioning stimulus, which also affects the outcome of ischemic postconditioning (Boengler et al., 2008). In general, pathological situations are often associated with reduced amounts of mitochondrial Cx43 and the preservation or increase of mitochondrial Cx43 helps to minimize the deleterious effects of such conditions. However, the precise role and regulation of mitochondrial Cx43 in pathological conditions of the heart or other organs remains to be investigated.

Whereas the aforementioned data relate to full-length Cx43, the N-terminally truncated form Gja1-20k is also present within mitochondria. Its impact on mitochondrial function, which may differ from that of full-length Cx43 given its localization in the outer mitochondrial membrane, under physiological conditions and in pathological situations especially in the setting of IR injury opens up a new perspective on the function of mitochondrial connexins.
